# In vitro targeting and selective killing of mcf-7 and colo320dm cells by 5-fluorouracil anchored to carboxylated SWCNTs and MWCNTs

**DOI:** 10.1007/s10856-021-06540-8

**Published:** 2021-06-14

**Authors:** Rutuja V. Kamble, Somnath D. Bhinge, Shrinivas K. Mohite, Dheeraj S. Randive, Mangesh A. Bhutkar

**Affiliations:** 1 Department of Pharmaceutical Chemistry, RCP, Kasegaon, Maharashtra 415 404 India; 2 Department of Pharmaceutics, RCP, Kasegaon, Maharashtra 415 404 India

## Abstract

The intention of the present work was to synthesize the f-MWCNT and f-SWCNT terminated with proper functional group, loading of 5-Flurouracil and to perform cytotoxic activity. Functionalization of MWCNTs and SWCNTs was achieved through the acid treatment (H_2_SO_4_ + HNO_3_). 5-flurouracil was loaded into the prepared functionalized CNTs, thereafter; in vitro drug loading capacity and % drug release were calculated. Also the prepared f-CNTs, 5-flurouracil loaded CNTs were distinguished by using SEM, TGA, DSC, X-ray diffraction, Raman and FTIR spectroscopy. MCF-7 and COLO320DM cells were treated with selected concentrations of 5-FU loaded f-MWCNTs and f-SWCNTs to estimate the cytotoxic activity. It was observed that 5-FU loaded f-SWCNTs showed good activity against selected cell lines than others. Moreover, apoptosis percentage was reported to be 84.46 ± 4.3515 and 92.78 ± 2.6549 for 5-FU loaded f-SWCNTs against MCF-7 and COLO320DM cells respectively. It is evident from the results that the prepared drug loaded CNTs have comparable antitumor activity in cancer cell lines.

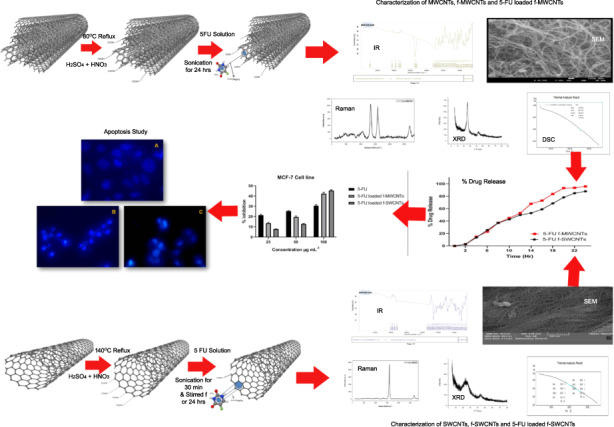

## Introduction

According to the report by WHO, breast, colon, liver, stomach, and lung cancer are the majority types of cancers, claiming lives of several cancer patients worldwide [1]. Breast cancer is the commonest type of cancer affecting around 21,00,000 women each year of which around 6,27,000 patients lose their life [2].

Localized tumors can be easily recovered with surgery; however, most of the aggressive cancers are metastasized and develop resistance to chemotherapy [3]. Therefore, the line of treatment is to use the fixed-dose combinations to inhibit the growth of cells and also to enhance the potential of their cytotoxic effect. Unfortunately, the toxicities of combination drug therapy is high to the patients owing to an increase in the drug numbers in the formulation [4]. Moreover, the cost of the anticancer agent(s) is very much high and ultimately, an increase of drug number, will lead to an increase in the cost of the formulation. Paclitaxel and 5-FU can be quoted as a classic example of fixed drug combination used in chemotherapy imparting good anticancer activity but unfortunately, they are interfering with each other [3, 5]. In addition, anticancer drugs pose a major issue with the solubility; often in aqueous medium owing to their hydrophobic nature [6]. To overcome the aforesaid disadvantages, several attempts have been made to develop formulations comprising of anticancer agents encapsulated in nanomaterials [6]. González-Lavado et al. [3] have reported that 5-FU loaded in nanomaterial (MWCNTs) exhibited good therapeutic effect against the human and murine cancer cells [3].

Interestingly, the ideal drug therapy involved with the targeted delivery and selective controlled release to optimize the therapeutic efficacy of the drug and to subsequently minimize its systemic toxicity [7]. In the entire world, Carbon is a dynamic element with their special arrangement and possessing a wide range of properties. For over 6000 years, carbon has been used for the reduction of metal oxides [8]. The different forms of carbon, namely graphite and diamond also belong to the family of chemical elements, and are in rolled layer(s) of graphite structure called carbon nanotubes (CNTs) [9, 10]. According to the layers the types of CNTs are classified as single-walled (SWCNTs) and multi-walled (MWCNTs) CNTs [11]. Moreover, CNTs exhibit wide range of biomedical applications owing to their extraordinary physio-chemical properties [12, 13]. Regrettably, CNTs have major technical impediment with their solubility which resulted in the lower biocompatibility, gastrointestinal absorption, blood transportation, and secretion in spite of being an excellent nanomaterial. Therefore, to overcome this disadvantage, Foldvari and Bagonluri [14]. have projected two fundamental elements namely functionalization/modification and dispersion (biomolecular, surfactant-assisted and solvent dispersion) of CNTs [14]. Modification of the surface CNTs i.e., functionalization is a lucrative option for increasing the hydrophilic nature of CNTs. f-CNTs present excellent interaction properties between biological sites and act as target carriers in drug delivery systems, especially as anticancer treatments [15, 16]. A broad range of pharmacotherapeutic agents could be simply conjugated with the f-CNTs due to an easy surface modification [17]. As per the report of Son et al. [18] functionalized CNTs have been used as nanocarriers to transport anticancer drugs, genes, and proteins for chemotherapy [18]. Some researchers have loaded 5-FU in CNTs using numerous techniques, and also proved their anticancer potentials [3, 19–23]. However, none of the researcher has attempted a study of synthesis, characterization and cytotoxic activity of 5-FU loaded f-MWCNTs and 5-FU loaded f-SWCNTs along with comparative data.

Thus, the present work was undertaken with an intention of synthesizing the f-MWCNT and f-SWCNT terminated with COOH groups, loading of 5-FU into the functionalized nanomaterial (MWCNTs and SWCNTs) and to characterize and perform a comparative study of their cytotoxic activity.

## Materials and methods

### Experimental

Highly pure (>99%) MWCNTs and (>85%) SWCNTs were procured from AD-nanotechnology Pvt. Ltd., Shimoga, Karnataka 577222. A gift sample of 5-fluorouracil was received from the Divi’s Laboratory, Mumbai. All other AR grade chemicals were used for the entire study. The UV and IR spectra were recorded on a UV Spectrophotometer (Jasco V630, Japan) and FTIR spectroscopy (Jasco 4100, Japan) respectively. The solutions were stirred with magnetic stirrer (Remi Instruments, Mumbai). Dissolution test apparatus USP II (Lab India DS8000) was used for the analysis of drugs. The characterization of the prepared samples of CNTs was carried out using Scanning Electron Microscopy (SEM) (VEGA3 TESCAN), XRD (Brucker D2 X-Diffractometer), TGA (Shimadzu DTG-60) and Raman Spectroscopy (EZ Raman Model EZN B532). Breast cancer cell namely MCF-7 and COLO320DM was procured from the NCCS (National Center for Cell Sciences), Pune—411007.

### Selection of the method for the functionalization of MWCNTs and SWCNTs

For COOH group functionalization of MWCNTs and SWCNTs, different types of techniques are available, amongst which base treatment, acid treatment and combined acid treatment are commonly used. The selection of method for the functionalization of MWCNTs and SWCNTs was based on the results of the dispersion stability study. f-MWCNTs and f-SWCNTs (5 mg) were treated with acid (HCl) and its combination (HCl + H_2_SO_4_). Thereafter, the treated CNTs were dispersed into 5 mL of phosphate buffer solution (pH 7.4) and sonicated for 15 min. Finally, the resultant solutions were kept in an air tight container for 15 days.

#### Functionalization of MWCNTs

100 mg pure MWCNTs were refluxed with 14M HNO_3_ and conc. H_2_SO_4_ (3:1) at 80 °C for continuous 18 h. Subsequently, the resultant solution was filtered using 0.1 mm PTFE membrane filter paper, then, the residue was washed with deionized water. Thereafter, the resultant solution was filtered under vacuum filtration, the f-MWCNT product was collected, and the f-MWCNTs were dried overnight at 80 °C under vacuum.

#### Functionalization of **SWCNTs**

100 mg pure SWCNTs was added to a solution containing H_2_SO_4_ and HNO_3_ in the proportion 3:1 and 500 mL of distilled water, the entire resultant solution was then sonicated for 4.0 h. Subsequently, the resultant solution was filtered under vacuum filtration. The f-SWCNT product was collected, and the f-SWCNTs were dried overnight at 80 °C under vacuum [24].

### Preparation of 5-fluorouracil drug loaded f-MWCNTs and f-SWCNTs

#### MWCNTs

100 mg of 5-fluorouracil was dissolved in 10 mL solution containing ethanol:water (1:9) [25]. Subsequently, 800 mg f-MWCNTs were added to this solution with continuous agitation using an ultra sonicator. Thereafter, the resultant mixture was stirred for 3 h, and the dispersion obtained was filtered using membrane filter paper (0.5 μm, Sigma Aldrich) equipped with vacuum filtration assembly. Finally, the residue was washed using ultra pure water. The final drug loaded f-MWCNTs were dried at 40 °C for 24 h [25]. Dry solid product was then stored until further use, whereas the collected filtrate containing unbound 5-fluorouracil was used for the estimation of 5-fluorouracil loading capacity of MWCNTs by using UV–Vis spectrophotometer. The collected filtrate containing unbound 5-fluorouracil was scanned at 280 nm, is the characteristic absorbance wavelength of 5-fluorouracil. Also calibration curve of 5-fluorouracil was accomplished with the parameter. Finally, obtained results were recorded to estimate the drug loading capacity as per Eq. 1; Tarawneh et al. [26].

1 $${\mathrm{Drug}}\,{\mathrm{loading}}\,{\mathrm{capacity}} \,=\, \frac{{{\mathrm{W}}_{{\mathrm{initial}}}5 - {\mathrm{fluorouracil}} \,-\, {\mathrm{W}}_{{\mathrm{unbound}}}5 - {\mathrm{fluorouracil}} \,\times\, {\mathrm{100}}}}{{{\mathrm{W}}_{{\mathrm{initial}}}5 - {\mathrm{fluorouracil}}}}$$

#### SWCNTs

Briefly, 100 mg of 5-fluorouracil was completely soluble in 800 mL of deionized water to get concentration at 0.125 mg mL^−1^. Then, 800 mg of f-SWCNTs were dispersed in a 5-fluorouracil solution. The resultant dispersion was sonicated for continuous 30 min (30 °C). Furthermore, the resultant dispersion was stirred continuously for 24 h in the dark with a stirrer. Consequently, the pH of the resultant dispersion was maintained at 4.0 to achieve optimum adsorption of 5-fluorouracil onto the f-SWCNTs. Furthermore, the resultant dispersion was centrifuged for next 15 min with 5000 rpm. It was then filtrated and rinsed with water. The rinsing procedure was followed with repeated four cycles, finally residue was oven dried at 65 °C and the resultant solid product was then stored in a air tight container till further use, whereas, collected supernatant having unbound 5-fluorouracil was used for the estimation of 5-fluorouracil loading capacity of SWCNTs by using UV–Vis spectrophotometer. The collected supernatant residue containing unbound 5-fluorouracil was scanned at 280 nm which is the characteristic absorbance wavelength of 5-fluorouracil. Also the calibration curve of 5-fluorouracil was plotted. Finally, obtained results were taken to estimate the drug loading capacity as per Eq. 1; Tarawneh et al. [26].

### Characterization of f-MWCNTs, f-SWCNTs, 5-FU loaded f-MWCNTs and 5-FU loaded f-SWCNTs

#### Fourier transforms infrared spectroscopy (FTIR)

The FTIR spectra of the MWCNTs, SWCNTs, f-MWCNTs, f-SWCNTs, 5FU loaded f-MWCNTs and 5FU loaded f-SWCNTs were recorded in the scale of 4200–400 cm^−1^.

#### Scanning electron microscopy (SEM)

Structure of the MWCNTs, SWCNTs, f-MWCNTs, f-SWCNTs, 5FU loaded f-MWCNT, and 5FU loaded f-SWCNT were confirmed by SEM and the images were captured by VEGA3 TESCAN Scanning microscope (Japan) and Jeol SEM (USA).

#### Raman spectroscopy

The radial breathing mode of CNTs is commonly used to evaluate their diameter by Raman spectroscopy. Degree of disorder of MWCNTs, f-MWCNTs, 5-FU loaded f-MWCNTs, SWCNTs, f-SWCNTs, and 5-FU loaded f-SWCNTs were determined. Side wall of the CNTs were structurally modified with an introduction of defects (sonication) or with the attachment of different chemical groups (functionalization) were also confirmed [26].

#### Differential scanning calorimeter (DSC)

DSC is employed to check the relative specific heat associated with transitions in CNTs, which help to provide qualitative and quantitative data about physical and chemical changes, involve exothermic and endothermic processes, or changes in heat capacity. Thermal properties of the MWCNTs, SWCNTs, f-MWCNTs, f-SWCNTs, 5FU loaded f-MWCNT, and 5FU loaded f-SWCNT were characterized by the DSC analysis [27].

#### X-Ray diffraction (XRD)

XRD analysis is analytical technique which is used for the phase characterization of crystalline materials [28]. Samples were scanned in the angular range of 100–600 in a Brucker D2 Phaser X-Diffractometer instrument. The f-MWCNTs, f-SWCNTs, 5-FU loaded f-MWCNTs, and 5-FU loaded f-SWCNTs were placed into instrument, X-ray passed through sample, and results were noted.

#### Thermo-gravimetric analysis (TGA)

TGA provides the details about chemical and physical properties namely sublimation, absorption, vaporization, adsorption, desorption, chemisorptions, dehydration, decomposition, and redox reactions [29]. The report of TGA represents the change in mass of CNTs substance is recorded as a function of time or temp. or upon heating a material, its weight increases or decreases. The plot of change in weight verses temperature known is as thermogram. Physical and chemical properties of the MWCNTs, SWCNTs, f-MWCNTs, f-SWCNTs, 5FU loaded f-MWCNT, and 5FU loaded f-SWCNT were characterized by the TGA analysis.

### In vitro drug release

Dialysis tubing method was followed for the in vitro drug release analysis of 5-FU from f-SWCNTs and f-MWCNTs containing drug in simulated gastric or an intestinal dissolution fluid [30], the required simulated fluid (pH 7.4, intestinal) was prepared in accordance with the procedure mentioned by Sobh et al. [23].

Before starting the experiments, dialysis bag/tubing having MW cutoff 12,000 Da and and 76 mm in size (Sigma Aldrich) was placed in the selected buffer solution for continuous 3.0 h [31]. Then, 1 mg of the samples was added into 3.0 mL of the dissolution media (buffer saline) containing dialysis tubing. Subsequently, sample containing tubing was kept in a closed receptor compartment containing dissolution media (50 mL, Buffer Saline) with stirring (100 rpm) at 37.0 ± 1 °C [31]. The buffer saline volume level was maintained during the experiment. Finally, 3 mL of drug loaded CNTs sample was withdrawn at regular time interval for UV analysis at the maximum absorption wavelength using the standard calibration curve method.

### Cytotoxic activity

MCF-7 and COLO320DM cells were separately treated with 5-FU loaded f-MWCNT and f-SWCNTs with selected concentrations at 25.00, 50.00, and 100.00 μg mL^−1^. Then the cells were CO_2_ incubated at a selected strength of 1 × 10^4^ cells mL^−1^ in the culture medium for continuous 1 day with 37.00 ± 2.00 °C. Consequently, 100 μL of 5-FU loaded f-MWCNTs and 5-FU loaded f-SWCNTs were dropped into the 96 well micro plates. Then the cells were again incubated for 24 h at 37.00 ± 1.00 °C with CO_2_ incubator. Further 20 μL MTT dye was added in a well, and then incubation was continued for continuous 4hr at aforesaid conditions. Lastly, 200 μL Dimethyl sulfoxide solution was added in the each well and kept for further 10 min. Elisa microplate plate reader was used to measure the absorbance at 550–570 nm in triplicate [18, 32].

### Cell morphology studies of 5-FU loaded f-MWCNTs and 5-FU loaded f-SWCNTs by DAPI staining

Microplate covered with cover slip (24 well) having a flat bottom was selected to study the cell morphology. MCF-7 and COLO320DM cells were separately seeded in a plate, further; the plate was incubated in a CO_2_ incubator with a 37.00 ± 1.00 °C temperature for an overnight. Subsequently, 100 μg mL^−1^ of 5-FU loaded CNTs (MWCNTs and SWCNTs) solution was added into the labeled well. After the addition of CNTs, plate was again incubated for continuous 1 day (24 h) at 37.00 ± 1.00 °C. Then, the PBS solution was used for washing purpose and the resultant solution was fixed with 4% formaldehyde for half an hour. Further, 20 µL of DAPI staining solution was added in well containing CNTs and incubated for continuous 5 min at ambient temperature in the dark condition. DAPI treated samples of control, 5-FU loaded MWCNTs and 5-FU loaded SWCNTs were examined under fluorescent microscopy.

### Statistical analysis

Statistical content of the drug release and cytotoxicity data was examined on Version 5.0, GraphPad Prism. All the cytotoxicity study experiments were conducted in triplicate. The obtained results were explored by using one-way ANOVA with standard error mean (SEM) differences were considered significant with a value as *p* < 0.05.

## Result

### Selection of the best method for the Functionalization of MWCNTs and SWCNTs

**Fig. 1:**
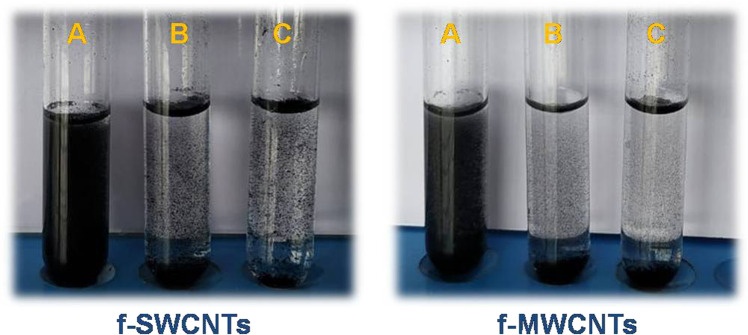
Dispersion of MWCNTs and SWCNTs after 15 days treatment with (**A**) combined acid (H2SO4 + HNO3), (**B**) plain acid (HCl), and (**C**) base

The results of the dispersion study (after 15 days) revealed that combined acid (H_2_SO_4_ + HNO_3_) treatment produce better dispersion (Fig. 1). Therefore, in the present study the acid treatment (H_2_SO_4_ + HNO_3_) was adopted for functionalization of MWCNTs and SWCNTs.

### Characterization of f-MWCNTs, f-SWCNTs, 5-FU loaded f-MWCNTs, and 5-FU loaded f-SWCNTs

#### FTIR analysis

**Fig. 2:**
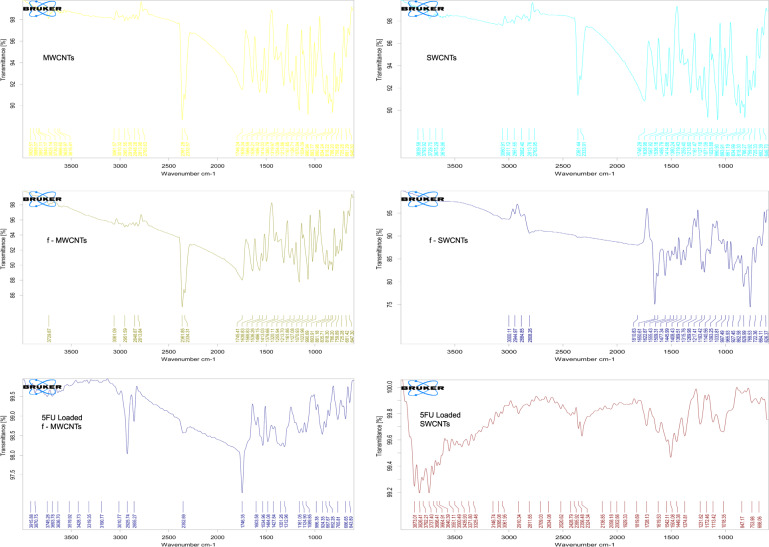
IR studies of MWCNTs, f-MWCNTs, 5-FU loaded f-MWCNTs, SWCNTs, f-SWCNTs, and 5-FU loaded f-SWCNTs

The functional group and band pattern of the MWCNTs, SWCNTs, f-MWCNTs, f-SWNTS, 5-FU, 5-FU loaded MWCNTs, and 5-FU loaded SWCNTs was confirmed using FT-IR (Fig. 2).

The IR spectra of MWCNT exhibited weak peaks at 818.22 and 1639.34 cm^−1^ corresponding to the bond stretching of C=C and C–C present in alkene group respectively. After the functionalization of MWCNTs and SWCNTs; C=O (carbonyl), C–O and O–H (hydroxyl) bond stretching vibration of COOH group were expected in the IR spectra. However, the IR spectra of f-MWCNTs showed the peaks for carbonyl, hydroxyl and C–O groups at 1745.41, 3061.09, and 1070.93 cm^−1^ respectively, which confirmed the functionalization of MWCNTs. Whereas, the IR spectra of 5FU loaded f-MWCNTs showed the peaks for carbonyl group present in the drug at 1746.35 and 1534.96 cm^−1^. In addition, hydroxyl, C–O, C–C, C–N, and N–H groups present in 5-FU were noted at 3010.77, 1089.85, 1351.26, 986.38, and 3319.35 cm^−1^ respectively.

The IR spectra of SWCNT exhibited weak peaks at 834.99 and 1638.98 cm^−1^ which is characteristic to the bond stretching of the C=C bending and C–C in alkene group respectively (Fig. 2). Whereas, the IR spectra of f-SWCNTs showed the peaks for carbonyl, hydroxyl and C–O groups at 1650.61, 3000.11, and 1193.42 cm^−1^ respectively. Thus, the graph of hydroxyl, carbonyl and C–O confirmed functionalization of MWCNTs. The IR spectra of 5-FU loaded f-SWCNTs showed the peaks for carbonyl group at 1728.13 and 1504.08 cm^−1^. Hydroxyl, C–O, C–C, C–N, and N–H groups were noted at 3146.74, 1113.42, 1374.81, 1018.35, and 3551.11 cm^−1^ respectively. Thus, graph of hydroxyl, carbonyl, and C–O thereby confirmed the functionalization of MWCNTs.

#### SEM analysis

**Fig. 3:**
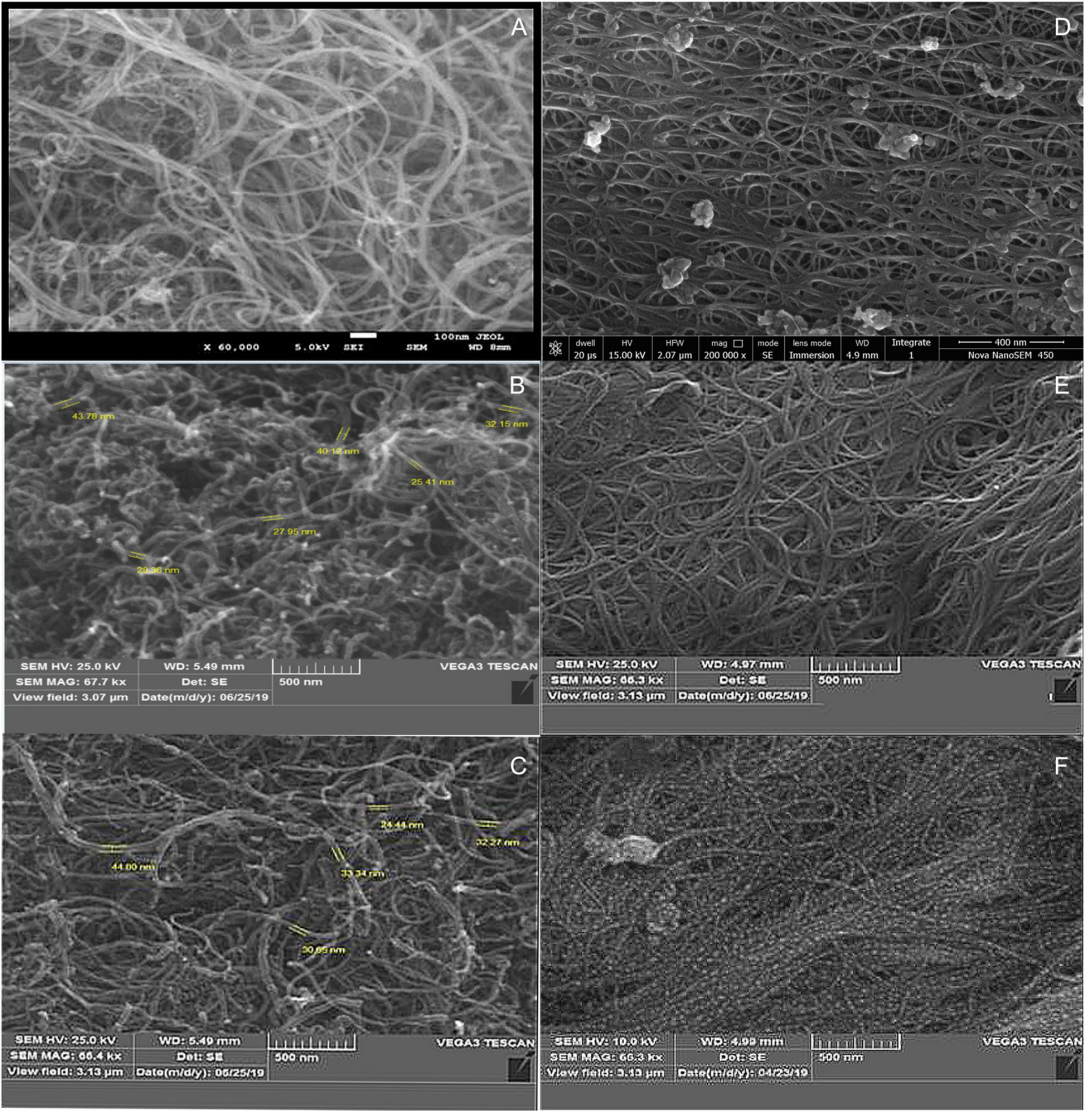
SEM analysis of (**A**) MWCNTs, (**B**) f-MWCNTs, and (**C**) 5-FU loaded f-MWCNs (**D**) SWCNTs, (**E**) f-SWCNTs, and (**F**) 5-FU

**Fig. 4:**
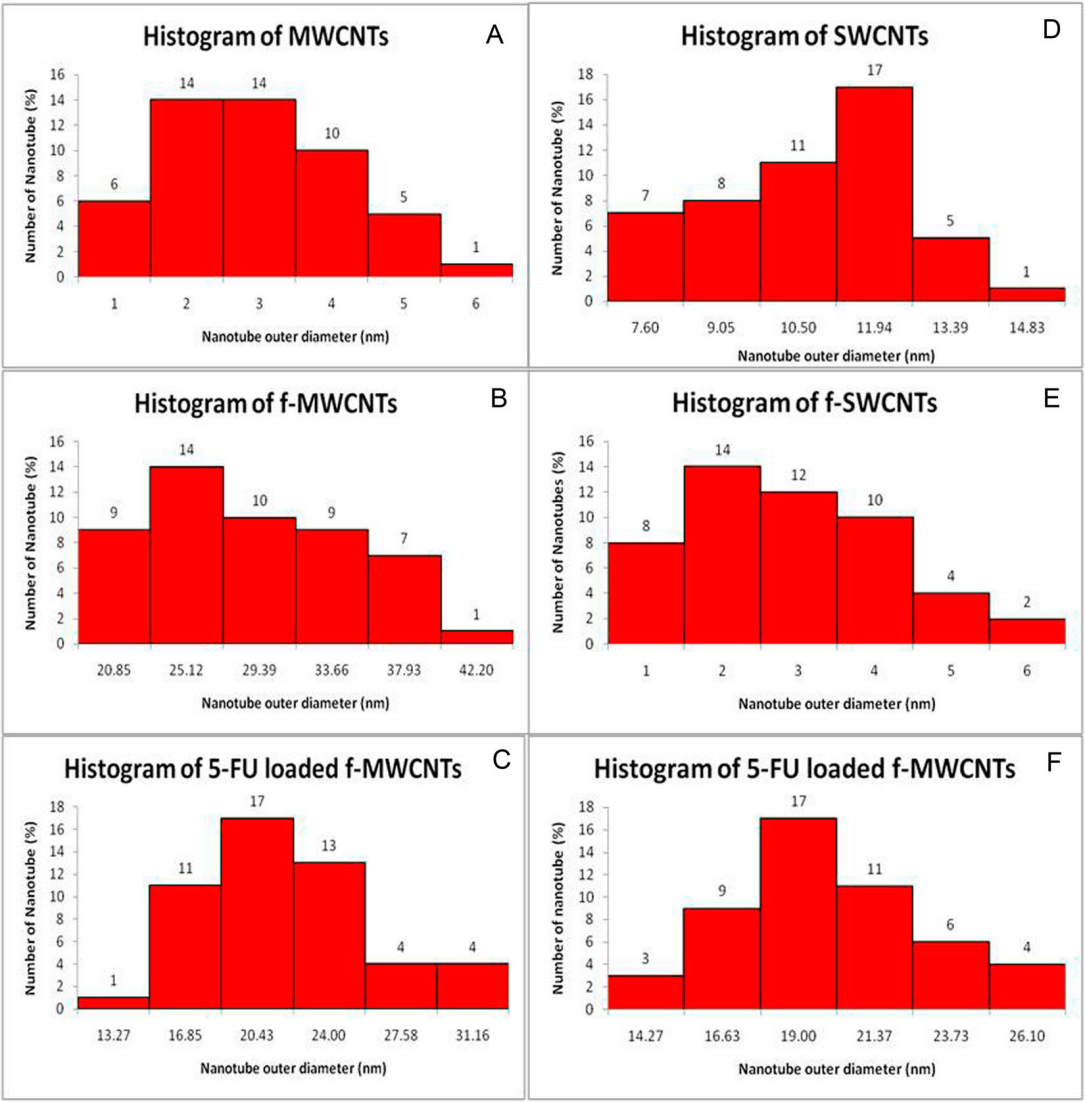
Histogram of (**A**) MWCNTs, (**B**) f-MWCNTs, and (**C**) 5-FU loaded f-MWCNs (**D**) SWCNTs, (**E**) f-SWCNTs, and (**F**) 5-FU loaded

The results of SEM exhibited the morphology of MWCNTs, SWCNTs, f-MWCNTs, f-SWCNTs, 5-FU loaded f-MWCNTs, and 5-FU loaded f-SWCNTs. As revealed in Fig. 3, craggy surface and rounded nanosheets of MWCNTs and SWCNTs were observed. Figure 3A, B, C revealed that MWCNTs, f-MWCNTs, and 5-FU loaded f-MWCNTs with an average size in the range of 18.53 ± 4.2790, 19.86 ± 4.5550, and 26.89 ± 6.1540 nm respectively, whereas, SWCNTs, f-SWCNTs, and 5-FU loaded f-SWCNTs were noted to be 12.08 ± 3.9720, 17.47 ± 3.5040, and 18.70 ± 3.0980 nm (Fig. 3D, E, F). Moreover, histograms of all CNTs are depicted in Fig. 4.

#### Raman spectroscopy

**Fig. 5:**
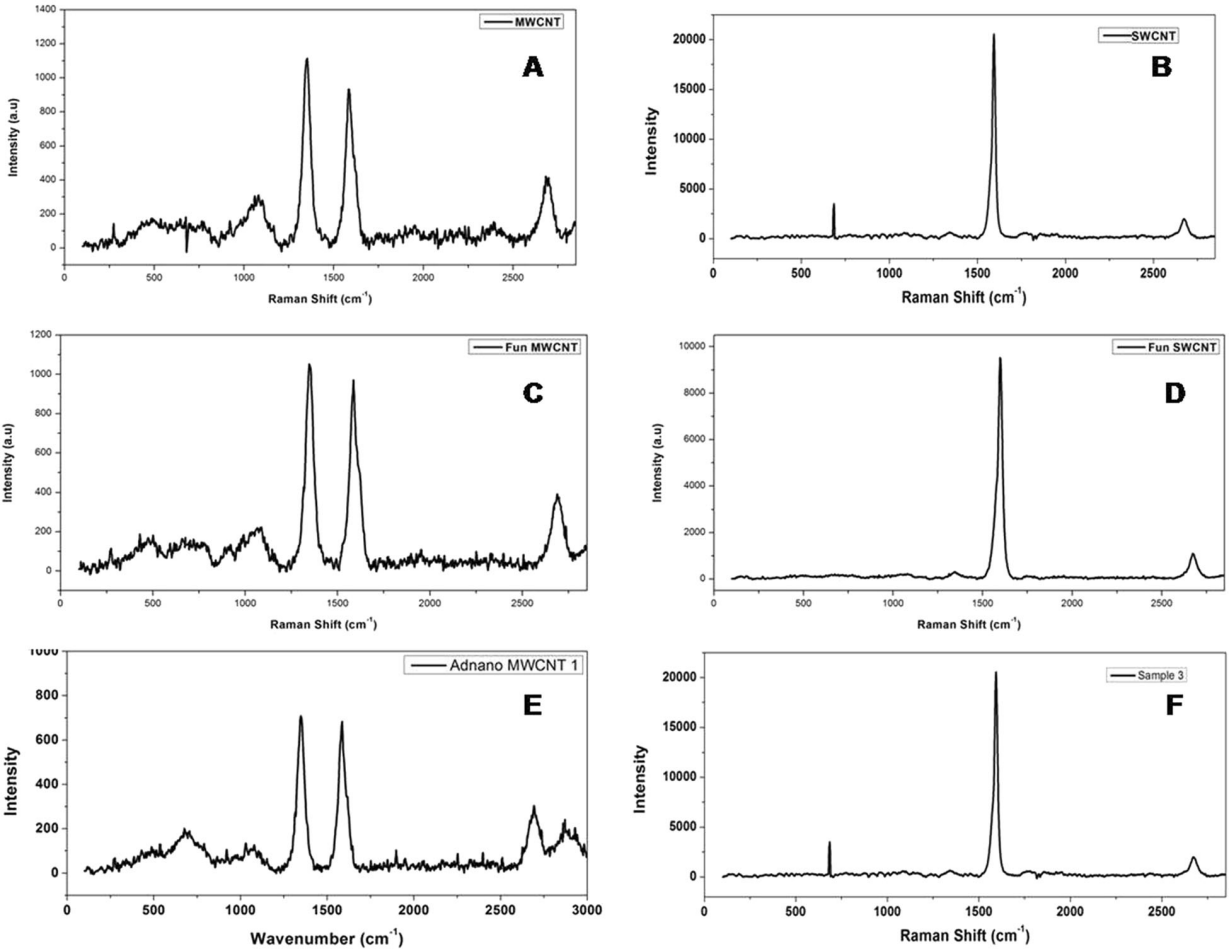
Raman spectroscopy of (**A**) MWCNTs, (**B**) SWCNTs, (**C**) f-MWCNTs, (**D**) f-SWCNTs, (**E**) 5-FU loaded f-MWCNTs, and (**F**) 5-FU loaded f-SWCNs

Degree of disorder of MWCNTs, SWCNTs, f-MWCNTs, f-SWCNTs, 5-FU loaded f-MWCNTs, and 5-FU loaded f-SWCNTs were confirmed by the Raman spectroscopy (Fig. 5). A Raman spectrum D and G band of MWCNTs and f-MWCNTs were noted at 1350 and 1595 cm^−1^. Disorder in the C (carbon) system and radial breathing form was identified with a D band at 1350 cm^−1^ and graphite form of graphene i.e., G band was noted at 1620 cm^−1^ in the Raman spectra of MWCNTs, f-MWCNTs, and 5-FU loaded f-MWCNTs. Zhao et al. [33]. have confirmed D band peak at 1341 cm^−1^ for disordered form of graphene and 1581 cm^−1^ for graphite form of grapheee [33]. A Raman spectrum D and G band of SWCNTs and f-SWCNTs were noted at 1330 and 1585 cm^−1^ respectively. Disorder in the carbon system was observed with a band D at 1330 cm^−1^ (weak peak) and graphite structure of graphene band G was noted at 1585 cm^−1^ (strong peak) in the Raman spectra of MWCNTs, f-MWCNTs, 5-FU loaded f-MWCNTs. The radial breathing mode (RBM) in MWCNTs and SWCNTs was observed at below the 300 cm^−1^ wave number [32, 34].

#### Differential scanning calorimeter (DSC)

**Fig. 6:**
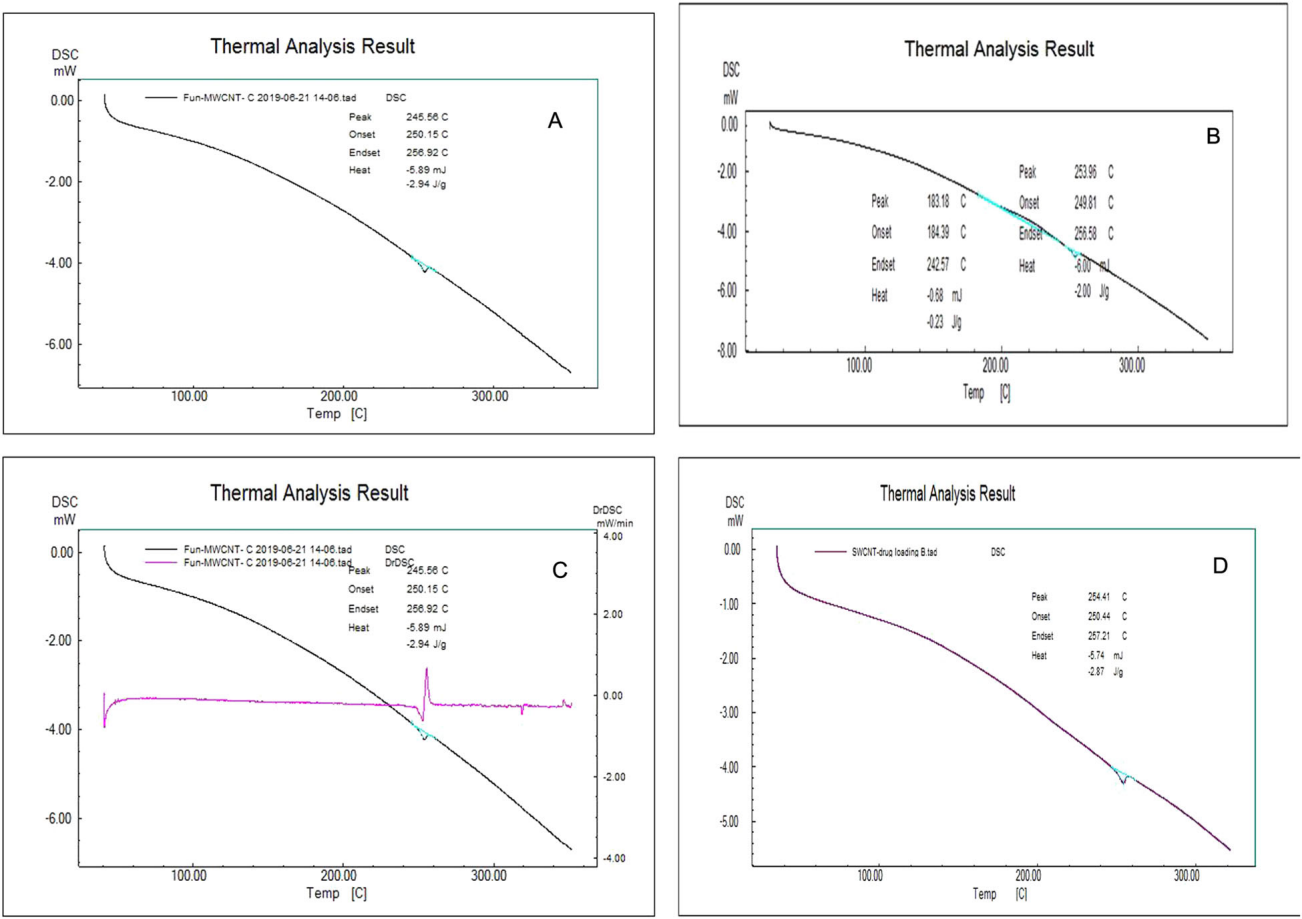
Differential scanning calorimeter (DSC) images of (**A**) f-MWCNTs, (**B**) f-SWCNTs, (**C**) 5-FU loaded f-MWCNTs, and (**D**) 5-FU loaded f-SWCNTs

DSC curves for f-MWCNTs and 5-FU loaded f-MWCNTs are depicted in Fig. 6A, C. The f-MWCNTs and 5-FU loaded f-MWCNTs noted sharp exothermic peak at 245.56 °C, corresponding to the melting transitions temperature and decomposition of MWCNTs. Sharp endothermic DSC peak signifies that MWCNTs were in pure crystalline state. In case of f-MWCNTs the DSC pattern of 5-FU loaded f-MWCNTs, indicated that most of the 5-FU was uniformly dispersed in CNTs at molecular level.

DSC curves for f-SWCNTs and 5-FU loaded f-SWCNTs are depicted in Fig. 6B, D. The f-SWCNTs and 5-FU loaded f-SWCNTs noted sharp exothermic peak at 254.98 °C, corresponding to the melting transitions temperature and decomposition of SWCNTs. Sharp endothermic DSC peak signifies that MWCNTs used was in pure crystalline state. In case of f-SWCNTs the DSC pattern of 5-FU loaded f-SWCNTs, which indicate the most of drug was uniformly dispersed in CNTs at the molecular level.

#### X-Ray diffraction (XRD)

**Fig. 7:**
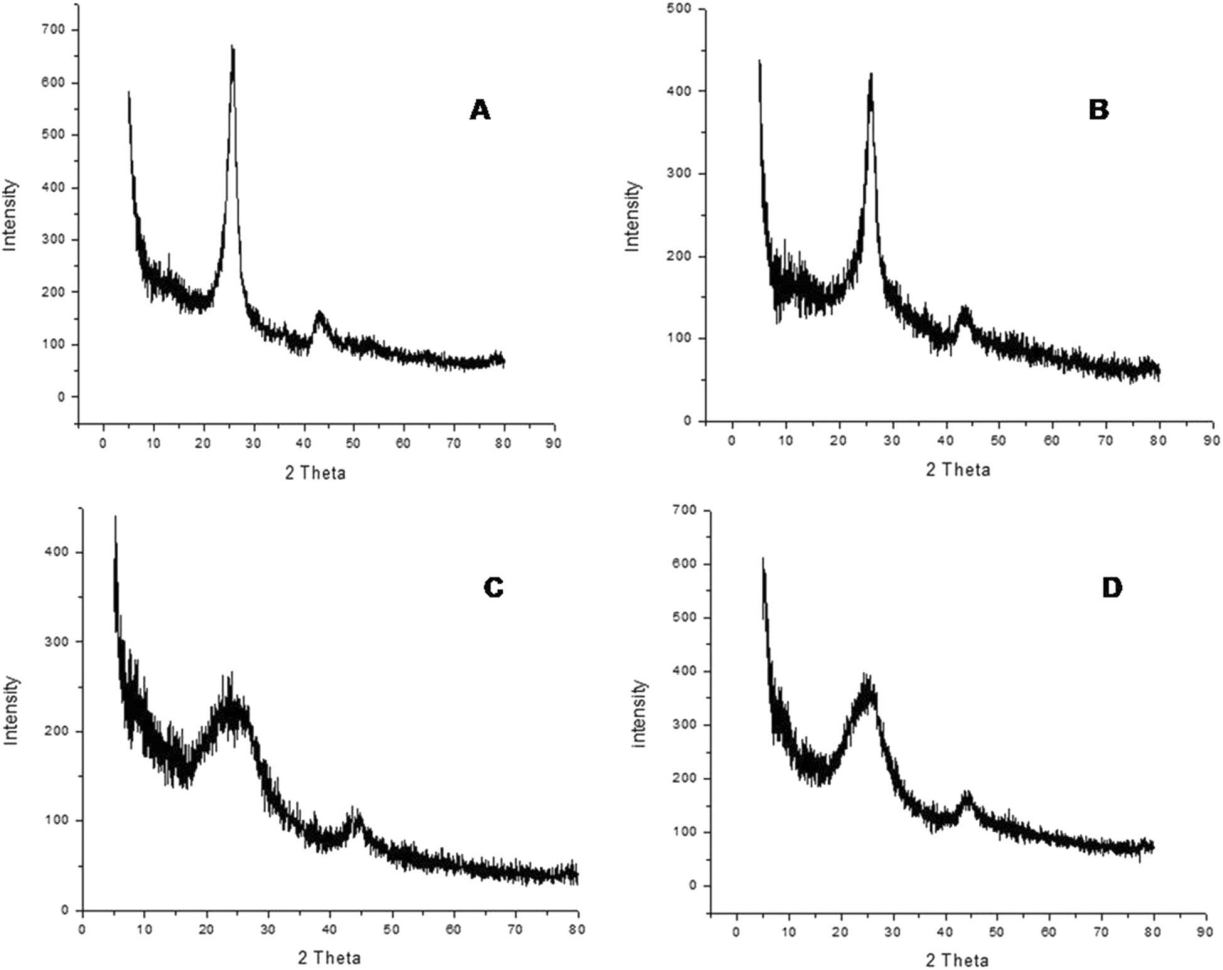
X-ray diffraction images of (**A**) f-MWCNTs, (**B**) f-SWCNTs, (**C**) 5-FU loaded f-MWCNTs, and (**D**) 5-FU loaded f-SWCNTs

XRD analysis of f-MWCNTs, f-SWCNTs exhibited two broad diffraction patterns with 2θ values of 25.0° and 45.0°. For 5-FU loaded f-MWCNTs, the leading peak at 2θ = 43.00° which is typical peak with an intense and sharp form was observed for f-MWCNTs, which indicated good crystallinity of 5-FU loaded MWCNTs composite. It was also observed that after loading of drug in f-MWCNTs and f-SWCNTs composite, crystallinity was noted with evidence of new varietal and low-intensity peaks (Fig. 7A, B, C, D).

#### Thermo-gravimetric analysis (TGA)

TGA/DTA was executed at a scanning range from 30 to 600 °C under N_2_ gas with heating rate of 20 °C min^−1^. Inflection temperatures of COOH functionalized SWCNTs were 480, 260, and 60 °C, and the corresponding mass changes occurred at −1.5%, −1.1%, and −1.9%, respectively, as shown in the DTG curves.

**Fig. 8:**
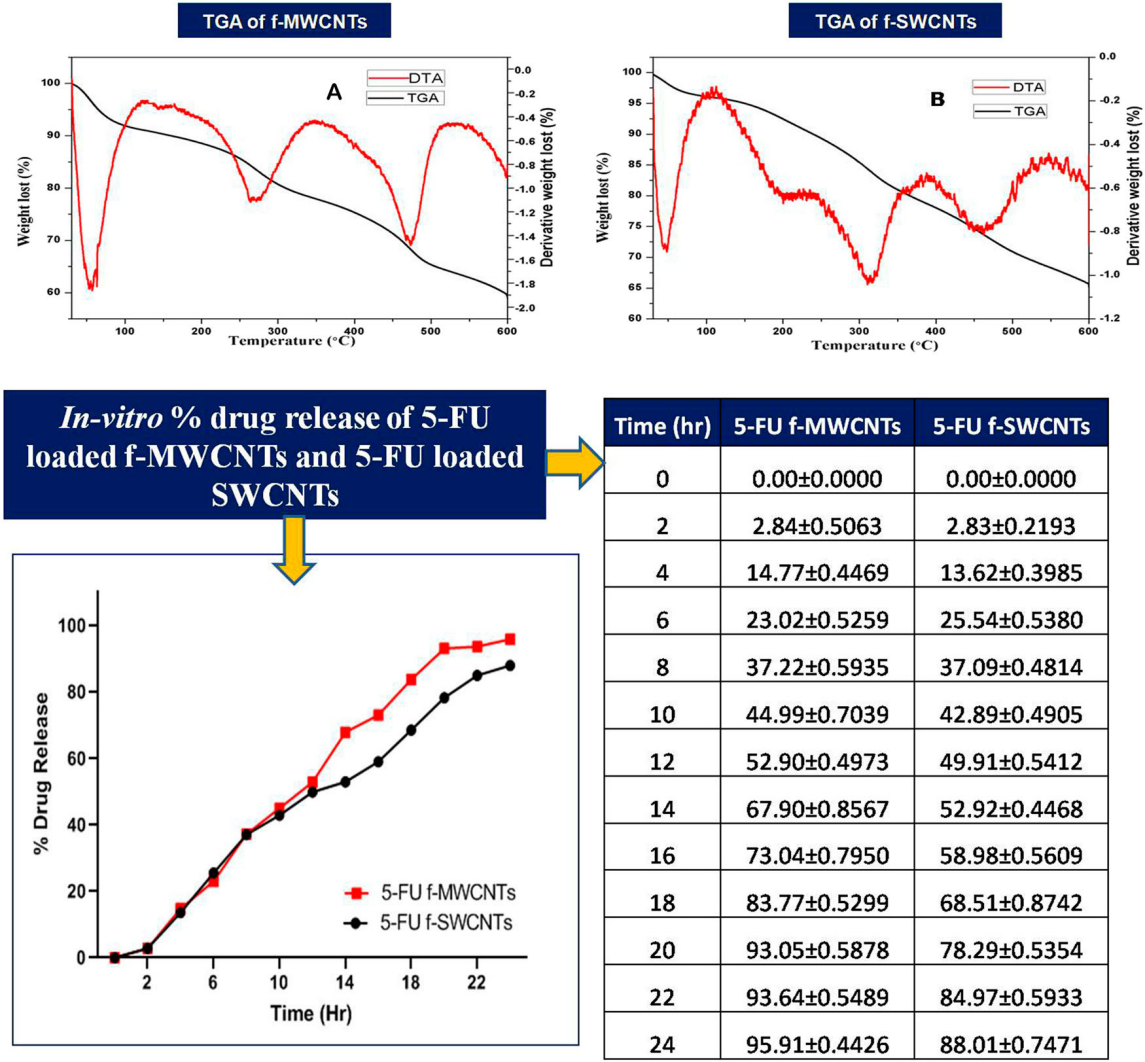
Thermo-gravimetric analysis (TGA) images of (**A**) f-SWCNTs and (**B**) 5-FU loaded f-SWCNTs; and In vitro % Drug release of 5-FU loaded f-MWCNTs and 5-FU

Whereas the inflection temperatures of 5-FU loaded f-SWCNTs were observed to be 470, 260, and 50 °C and the corresponding mass changes occurred at −0.7%, −1.1%, and −0.8%, respectively, as shown in the DTG curves. Figure 8 showed the differences in the total mass of the f-SWCNTS and 5-FU-SWCNT samples corresponding to the drug.

### The loading rate of 5-FU onto f-MWCNTs and f-SWCNTs

The drug loading rate is the vital part of the targeted drug delivery approach. 5-FU entrapment efficiency was found to be significantly higher for f-MWCNTs and f-SWCNTs than MWCNTs and SWCNTs at different ratios of the CNTs:5-FU. The 5-FU entrapment efficiency was studied at different ratio of the f-MWCNTs:5-Fu; f-SWCNTs:5-FU. The starting ratio of CNTs (f-SWCNTs/f-MWCNTs):5-FU were 1:0.5(w/w), which increased the concentration of 5-FU only upto 1:4 (w/w). The 5-FU entrapments in CNTs were quantitatively noted at 280 nm by UV–Vis spectroscopy. 5-FU entrapments were observed to be 95.83 ± 2.57% and 93.43 ± 1.65 in f-MWCNTs and f-SWCNTs respectively. 5-FU is the aromatic heterocyclic compound, due to unique property π-π staging and hydrophobic interactions possible in side wall of CNTs and the chances of increasing the entrapment of 5-FU in CNTs. 5-FU is pyrimidine analog containing C=O and N–H group. Thus, the present COOH and OH group in the f-MWCNTs and f-SWCNTs form strong hydrogen bond with C=O and NH group.

### In vitro drug release of 5-FU loaded MWCNTs

Phosphate buffer solution with pH 7.4 was used as dissolution media for conducting in vitro drug release. The drug release profiles of 5-FU loaded f-MWCNTs and f-SWCNTs are shown in Fig. 8. For 5-FU loaded f-MWCNTs and 5-FU loaded f-SWCNTs, the drug release rate was observed to be 44.98 ± 0.7038 and 42.89 ± 0.4904 (at pH 7.4) respectively after 10 h, whereas, 95.90 ± 0.4425 and 88.00 ± 0.7471% (at pH 7.4) was noted for 5-FU loaded f-MWCNTs and 5-FU loaded f-SWCNTs after 24 h, respectively (Fig. 8). 5-FU loaded f-MWCNTs and f-SWCNTs illustrated an initial burst release, attributed to the 5-FU loosely anchored to the walls of CNTs or held within the CNTs.

### Cytotoxicity study

Cytotoxicity study was carried out with MTT proliferation assay as previously described by Bhinge et al. 2020 [35], to confirm the sensitivity of MCF-7 and COLO320DM cells against 5-FU (bulk form), 5-FU loaded f-MWCNTs and 5-FU loaded f-SWCNTs. MTT assay setup was executed for the inhibition of MCF-7 and COLO320DM cell proliferation. The selected cell line was treated with various strength solutions of 5-FU (bulk), 5-FU loaded f-MWCNTs and 5-FU loaded f-SWCNTs.

**Fig. 9:**
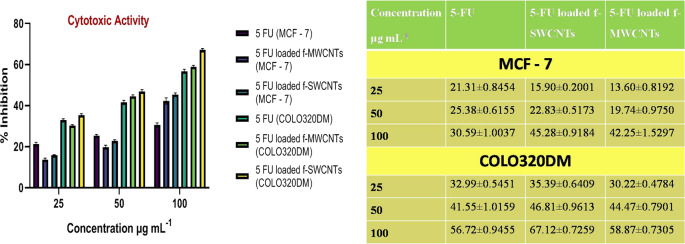
Cytotoxic activity of 5-FU, 5-FU loaded MWCNTs, and 5-FU SWCNTs against the MCF-7 and COLO320DM cell line

In this study, cell viability was confirmed after addition of 25.00, 50.00, and 100.00 μg mL^−1^ solution of 5-FU, 5-FU loaded f-MWCNTs, and 5-FU loaded f-SWCNT after exact 50 h. The inhibition rates were noted to be 30.59 ± 1.0037, 45.28 ± 0.9184, and 42.25 ± 1.5297% for 100.00 μg mL^−1^ of 5-FU, 5-FU loaded f-SWCNTs, and 5-FU loaded f-MWCNTs respectively against MCF-7 cell lines. While, % inhibition was to be noted at 56.72 ± 0.9455, 67.12 ± 0.7259, and 58.87 ± 0.7305% at a concentration of 100 μg mL^−1^ for 5-FU, 5-FU loaded f-SWCNTs, and 5-FU loaded f-MWCNTs respectively against the COLO320DM cells (Fig. 9).

### Cell morphology studies of 5-FU loaded f-MWCNTs and 5-FU loaded f-SWCNTs by DAPI staining for fluorescence microscopy

Morphology changes in MCF-7 and COLO320DM cells were identified with DAPI staining approach with the treatment of 5-FU loaded f-MWCNTs and 5-FU loaded f-SWCNTs.

**Fig. 10:**
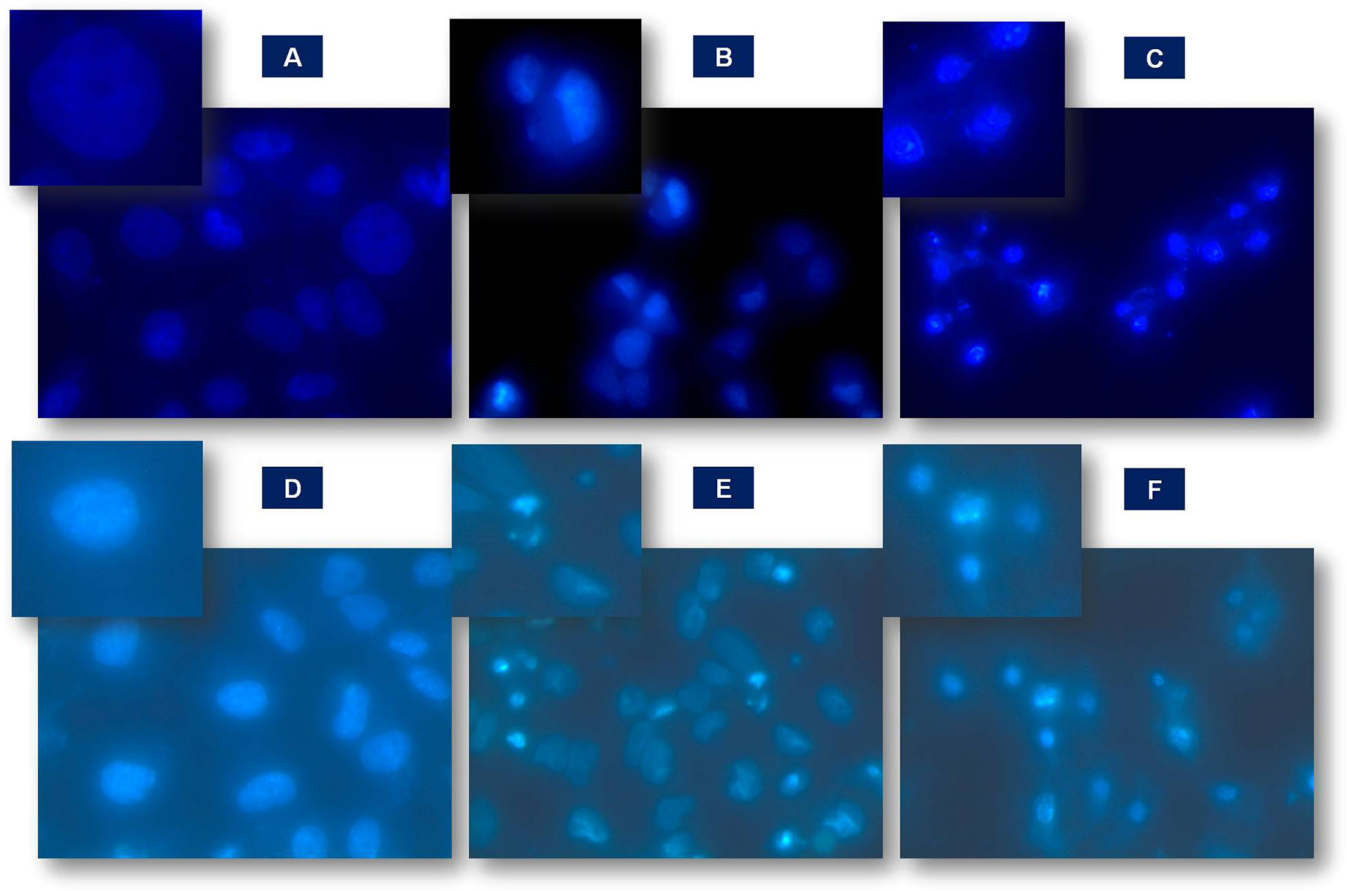
Apoptosis activity of (**A**) Control of MWCNTs), (**B**) 5-FU loaded MWCNTs, and (**C**) 5-FU SWCNTs against the MCF-7 cell line; and (**D**) control of SWCNTs, (**E**) 5-FU loaded MWCNTs and (**F**) 5-FU SWCNTs against the COLO320DM cell line

An image of the fluorescence microscopy for control treated and cell treated with 5-FU loaded f-MWCNTs, and 5-FU loaded f-SWCNTs are depicted in Fig. 10. The control exhibited normal intact nuclei of the cells with weak homogenous blue staining pattern. On the contrary, the cells treated with 5-FU loaded f-MWCNTs and 5-FU loaded f-SWCNTs were noted as smaller nuclei with bright chromatin condensation, by blebbing, nuclear fragmentation, and apoptotic bodies means smaller spherical fragments formation. Apoptosis percentage was reported to be 4.22 ± 1.86, 41.72 ± 3.56, and 84.46 ± 4.35 for control, 5-FU loaded f-MWCNTs, and 5-FU loaded f-SWCNTs, respectively, against the MCF-7 cells. Whereas, in the COLO320DM cells the % inhibition was observed to be 2.65 ± 2.0123, 45.58 ± 2.0925, and 92.78 ± 2.6549 for control, 5-FU loaded f-MWCNTs and 5-FU loaded f-SWCNTs respectively (Table 1). The results clearly indicated that 5-FU loaded MWCNTs and 5-FU loaded SWCNTs induced apoptosis in MCF-7 and COLO320DM cells when compared with control (Fig. 10).

**Table 1:** Apoptosis study of control, 5-FU loaded f-MWCNTs, and 5-FU loaded f-SWCNTs

Concentration μg mL^−1^	Control	5-FU loaded f-SWCNTs	5-FU loaded f-MWCNTs
**MCF-7**			
**100**	4.22 ± 1.8617	84.46 ± 4.3515	41.72 ± 3.5603
**COLO320DM**			
**100**	2.65 ± 2.0123	92.78 ± 2.6549	45.58 ± 2.0925

## Discussion

Conventional drug delivery system is often confronted with the problem of poor site specificity which may lead to unwanted effects of the drugs; especially in the case of anticancer therapies. In the effective treatment of tumors it is vital that the drug should target the tumor site in optimum concentration, which can be achieved by use of specific carriers. Therefore, it’s an urgent need to find out a suitable carrier for the anticancer drug.

CNTs are considered as a promising tool for the delivery of various therapeutic molecules owing to its small size and high specific surface areas which facilitates their adsorption on biological substrates [18]. CNTs are known to increase the cell permeability in cancer cells [18].

Moreover, due to effective interaction properties between biological site(s), CNT’s are considered to be excellent target carriers in drug delivery systems, especially for cancer treatments [15, 16]. MWCNTs and SWCNTs exhibit wide ranges of physical properties. Plain CNTs are hydrophobic in nature and exhibit less interaction toward the targeted site, however, modification of the surface CNTs i.e., functionalization is a lucrative option for increasing the hydrophilic nature of CNTs which will contribute to maximum chemical binding of drug moieties thereby increasing the drug concentration in the CNTs. Thus, in the present study the CNTs have been functionalized with acids to anchor the –COOH group at the side wall of the CNTs and thereafter, the drug under study was anchored with the modified CNTs–COOH.

Pyrimidine analogs have been commonly used in cancer treatments and the most common derivative namely; 5-FU is an example of this class which could treat several solid tumors like liver, rectal, colon, pancreatic, ovarian, breast, gastric, and bladder cancer etc. However, 5-FU poses the problem of poor absorption and has been reported with severe side effects. Therefore, it is necessary to increase its site specific action which will facilitate its effective absorption along with fewer side effects. An attempt was thus made in the direction to incorporate 5-FU into the CNTs as discussed earlier.

González-Lavado et al. [3]. clearly stated that 5-FU was loaded into plain CNTs via π-stacking onto the side wall of MWCNTs [3]. However, plain CNTs possess less disperse properties and less stability for loading of 5-FU. Therefore, in the present study we have employed f-CNTs instead of plain CNTs. The probable mechanism involved may be in accordance with the mechanism stated by González-Lavado et al. [3]. Also, another possibility is an interaction of NH group of 5-FU with COOH group of f-CNTs with liberation of water and the said fact is depicted in IR spectrum of 5-FU loaded f-MWCNTs and 5-FU loaded f-SWCNTs wherein, the peak of C–O group is not observed, however in IR spectra of f-MWCNTs and f-SWCNTs the group peaks are clearly seen (Fig. 2). Moreover, Fig. 8 depicts the TGA study where about 8% of the total mass of f-SWCNT sample corresponds to 5-FU loaded f-SWCNT.

In the present study the CNTs were functionalized with the mixture of H_2_SO_4_ + HCl, which was selected based on the revealed results of the dispersion study for 15 days, which have been earlier reported. Moreover, all the synthesized CNTs were characterized using hyphenated tools viz; FTIR, SEM, Raman, DSC, XRD, and TGA analysis. The aforesaid techniques revealed the morphological properties of CNTs and drug moiety. 5-FU entrapment was observed to >90% for both CNTs due to may be π–π staging and hydrophobic interactions on side wall of MWCNTs and SWCNTs as discussed earlier. Also, the % drug release of 5-FU loaded f-MWCNTs and 5-FU loaded f-SWCNTs exhibited control release pattern over a period of 24 h as compared to pure 5-FU, therefore, the system will be beneficial to achieve targeted as well as controlled drug delivery. Importantly, the prepared CNTs were evaluated for the in vitro anticancer activity. It was observed that the drug loaded f-SWCNTs exhibited better % inhibition rate than the 5-FU loaded f-MWCNTs and plain 5-FU against MCF-7 and COLO320DM cell line. 5-FU loaded f-MWCNTs and 5-FU loaded f-SWCNTs showed better percent inhibition than the 5-FU may be due to cell permeability nature of the CNTs [18]. Furthermore, probable mechanism was verified with apoptosis studies, our study revealed that all studied parameters exhibited almost same results for both CNTs, however in apoptosis study 5-FU loaded f-SWCNTs showed the maximum apoptotic cell (84.46 ± 4.35%), as compared to 5-FU loaded f-MWCNTs (41.72 ± 3.56). Based upon the said facts, the 5-FU loaded f-MWCNTs and 5-FU loaded f-SWCNTs can enhance the uptake of 5-FU in MCF-7 and COLO320DM cells. This is presumably due to the high aspect ratio and the high surface area of the CNTs. In this research, we have tried to explore the comparison between 5-FU loaded f-MWCNTs and 5-FU loaded f-SWCNTs anticancer activity along with % of apoptosis, which was not focused in earlier studies. Also, there is further scope to explore more research on CNTs coated exteriorly with biocompatible layers, e.g., PEG, polysaccharide, protein, and DNA to study their effect on the efficacy of coated CNTs which have not been a scope of our study. The results and findings of the present investigation will be helpful and provide an insight on coating of CNTs exteriorly with suitable biocompatible polymers.

## Conclusion

MWCNTs and SWCNTs have been considered as lucrative options for the targeted and controlled drug delivery owing to their intrinsic properties. As per our intention, we have functionalized both CNTs (single and multi walled) and loaded 5-FU drug into the f-CNTs. Thereafter, the drug loaded CNTs were characterized using advanced tools and studied for their anticancer potential against MCF-7 and COLO320DM cell lines. The drug loaded f-SWCNTs exhibited excellent % cell inhibition than the 5-FU. Moreover, apoptosis study proved that the MCF-7 and COLO320DM cells treated with 5-FU loaded MWCNTs and 5-FU loaded SWCNTs exhibited smaller nuclei size with a bright chromatin condensation, blebbing, nuclear fragmentation, and formation of apoptotic bodies. With these obtained results, it can be concluded that the prepared drug loaded CNTs possess significant comparable antitumor activity in breast cancer cell lines thus, providing a hopeful technique to enhance the efficacy of traditional chemotherapies. Furthermore, CNT’s may prove to be immensely beneficial owing to its diverse properties in effective management of cancer therapy.

## Supplementary information

Prime Novelty Statement
