# Supplementary material for: In vitro targeting and selective killing of mcf-7 and colo320dm cells by 5-fluorouracil anchored to carboxylated SWCNTs and MWCNTs
Source: J Mater Sci Mater Med. 2021 Jun 14;32(6):71. doi: 10.1007/s10856-021-06540-8 (PMC8203503; doi:10.1007/s10856-021-06540-8)
Supplement: Supplementary file 1 — Prime Novelty Statement [file 10856_2021_6540_MOESM1_ESM.doc]

**Prime Novelty statement**

In this research, the authors have tried to explore anticancer activity of prepared 5-Fluro uracil loaded carboxylated CNTs (MWCNTs and SWCNTs) against COLO320DM and MCF-& human cell lines along with along with % of apoptosis, which was not focused in earlier literature collectively.
